# Comparative analysis of two navigation techniques based on augmented reality technology for the orthodontic mini-implants placement

**DOI:** 10.1186/s12903-023-03261-y

**Published:** 2023-08-05

**Authors:** Elena Riad Deglow, Álvaro Zubizarreta-Macho, Héctor González Menéndez, Juan Lorrio Castro, Agustín Galparsoro Catalán, Georgia Tzironi, Ana Belén Lobo Galindo, Luis Óscar Alonso Ezpeleta, Sofía Hernández Montero

**Affiliations:** 1 Department of Implant Surgery, Faculty of Health Sciences, Alfonso X El Sabio University, Avda Universidad, 1. 28691, Villanueva de La Cañada, Madrid, Spain; 2 Department of Surgery, Faculty of Medicine and Dentistry, University of Salamanca, 37008 Salamanca, Spain; 3 Department of Endodontics, School of Health Sciences, University of Zaragoza, Saragossa, Spain

**Keywords:** Orthodontics, Mini-implants, Orthodontic anchorage, Augmented reality

## Abstract

To analyze and compare the accuracy and root contact prevalence, comparing a conventional freehand technique and two navigation techniques based on augmented reality technology for the orthodontic self-drilling mini-implants placement.

**Methods** Two hundred and seven orthodontic self-drilling mini-implants were placed using either a conventional freehand technique (FHT) and two navigation techniques based on augmented reality technology (AR TOOTH and AR SCREWS). Accuracy across different dental sectors was also analyzed. CBCT and intraoral scans were taken both prior to and following orthodontic self-drilling mini-implants placement. The deviation angle and horizontal were then analyzed; these measurements were taken at the coronal entry point and apical endpoint between the planned and performed orthodontic self-drilling mini-implants. In addition, any complications resulting from mini-implant placement, such as spot perforations, were also analyzed across all dental sectors.

**Results** The statistical analysis showed significant differences between study groups with regard to the coronal entry-point (*p* < 0.001), apical end-point(*p* < 0.001) and angular deviations (*p* < 0.001). Furthermore, statistically significant differences were shown between the orthodontic self-drilling mini-implants placement site at the coronal entry-point (*p* < 0.0001) and apical end-point (*p* < 0.001). Additionally, eight root perforations were observed in the FHT group, while there were no root perforations in the two navigation techniques based on augmented reality technology.

**Conclusions** The navigation techniques based on augmented reality technology has an effect on the accuracy of orthodontic self-drilling mini-implants placement and results in fewer intraoperative complications, comparing to the conventional free-hand technique. The AR TOOTH augmented reality technique showed more accurate results between planned and placed orthodontic self-drilling mini-implants, comparing to the AR SCREWS and conventional free-hand techniques. The navigation techniques based on augmented reality technology showed fewer intraoperative complications, comparing to the conventional free-hand technique.

## Background

Anchorage in orthodontics is considered an important factor as it can prevent undesirable tooth movements created as a reaction from orthodontic forces applied to adjacent teeth [1]. The introduction of temporary anchorage devices (TAD) has drastically enhanced orthodontic treatments offering an alternative to conventional orthodontic treatments [2]. Mini-implants can be of stainless steel, titanium or titanium alloy and theirs dimensions are between 8 to 20 mm of length and 1 to 2 mm of diameter [1]. Their small size and smooth surfaces made them popular among clinicians over the past few years, allowing minis crews to be loaded immediately after their insertion. Since they are not osseointegrated they can easily be removed after treatment. Nevertheless, TADS ability to be maintained close to the adjacent bone, offers stability to the reactive forces resulting in minimized anchorage loss [3].However, complications can be occurred related to several variables, which may include inherent characteristics of the patient (age, gender, systematic diseases, periodontal status, smoking, skeletal pattern) [4], operator experience [5], mechanical properties of the orthodontic micro-screw [6], patient oral hygiene [7] etc. Placement characteristics such as placement site [8–10], insertion angle [11], root proximity [12], as well as bone characteristics related to bone density [13], bone stress [14], and orthodontic force applied [12, 13] also may play an important role to long- and short-term success of the mini-implant. In addition to that, root contact during insertion should be taken into consideration to avoid possible root damage to the teeth close to the operation site [7, 10, 12]. Cone-beam computed tomography (CBCT) scan [12], standard two-dimensional radiography [15], and panoramic radiography [16] are of great use in order assist in pre-operative planning. Additionally, to pre-operative radiographic examination, research indicates that guided procedures for implant placement have encouraging results especially when compared to conventional freehand implant placement [17]. A freehand procedure can be influenced by a number of factors and its accuracy has been shown to depend on the anatomical condition and the surgeon’s experience as well [18]. Therefore, computer-aided treatment approaches have been highlighted to provide safer, more conservative, and more accurate results as well as shorter treatment times, comparing to conventional free-hand techniques. In addition, computer-aided treatment approaches have demonstrated to improve postoperative, reduce morbidity, and helps the clinician to avoid intraoperative complications such as root perforation of the adjacent teeth, the nasal and maxillary sinus invasion, in comparison to the free-hand approach [19].

Augmented reality (AR) technology has been widely developed since it allows an accurate alignment of the digital files on a real environment as well as to track the registered object at real-time; specifically, AR technology may provide additional information to the clinician which can help the therapeutic procedure since the operator may visualize the digital therapeutic planning on the dental orography of the patient through an augmented reality device [20]. Therefore, the promising results of the AR technology has encouraged researchers and clinicians to apply the AR technology to other disciplines; however, there are only a limited number of in vitro and clinical studies available in the literature [20, 21].

The present study aims to analyze and compare the accuracy and root contact prevalence, comparing a conventional freehand technique and two navigation techniques based on augmented reality technology for the orthodontic self-drilling mini-implants placement. The null hypothesis (H_0_) states that there is no difference in the accuracy and root contact prevalence between a conventional freehand technique and two navigation techniques based on augmented reality technology for the orthodontic self-drilling mini-implants placement at the coronal entry-point, apical end-point and angular deviation in all dental sectors.

## Methods

### Study design

Researchers conducted a controlled experimental trial between October 2022 to January 2023 at the Dental Centre of Innovation and Advanced Specialties at Alfonso X El Sabio University in Madrid, Spain. The Ethical Committee of the Faculty of Health Sciences at Alfonso X El Sabio University approved the study in October 2022 (process no. 10/2022). In addition, this study was conducted in accordance with the principles outlined by the German Ethics Committee’s statement on using organic tissues for medical research (Zentrale Ethikkommission, 2003). All patients gave their informed consent for their teeth to be used in the study. A power of 80.00% was calculated using the bilateral Student’s *t-*test for two independent samples. When used to calculate the variation from the null hypothesis H_0_: μ_1_ = μ_2_, the significance level of 5.00% and power of 80.00% meant that 207 orthodontic self-drilling mini-implants were necessary for the purposes of this study.

### Experimental procedure

Upper teeth from all dental sectors, which required extraction due to periodontal and orthodontic reasons, were selected for study from cases treated at the Dental Centre of Innovation and Advanced Specialties at Alfonso X El Sabio University (Madrid, Spain), between April and October 2022. The teeth were placed in fourteen experimental models of epoxy resin (Ref. 20–8130-128, EpoxiCure®, Buehler, IL, USA) with 16 teeth each. A silicone splint was created by a conventional impression to a dental training model of acrylic resin, and the teeth were placed on it. Subsequently, the epoxy resin (Ref. 20–8130-128, EpoxiCure®, Buehler, IL, USA) was mixed following the manufacture recommendations and poured inside the silicone splint with the teeth. After the epoxy resin setting the silicone splint was removed from the epoxy resin model. The orthodontic self-drilling mini-implants(Dual Top® Anchor System, JEIL Medical Corporation, Guro-gu, Seoul, Republic of Korea) were randomly assigned (Epidat 4.1, Galicia, Spain) to one of the following study groups: Group A. Orthodontic self-drilling mini-implants placement in the incisive-canine sector by an augmented reality device (Hololens2, Redmond, WA, USA)based on tooth visualization (AR TOOTH-i) (*n* = 23), B. Orthodontic self-drilling mini-implants placement in the incisive-canine sector by an augmented reality device (Hololens2, Redmond, WA, USA)based on navigation guidance (AR SCREWS-i) (*n* = 23), C. Orthodontic self-drilling mini-implants placement in the incisive-canine sector by conventional freehand technique(FHT-I) (*n* = 23), Group D. Orthodontic self-drilling mini-implants placement in the premolar sector by an augmented reality device (Hololens2, Redmond, WA, USA)based on tooth visualization (AR TOOTH-p) (*n* = 23), E. Orthodontic self-drilling mini-implants placement in the premolar sector by an augmented reality device (Hololens2, Redmond, WA, USA)based on navigation guidance (AR SCREWS-p) (*n* = 23), F. Orthodontic self-drilling mini-implants placement in the premolar sector by conventional freehand technique(FHT-p) (*n* = 23), Group G. Orthodontic self-drilling mini-implants placement in the molar sector by an augmented reality device (Hololens2, Redmond, WA, USA)based on tooth visualization (AR TOOTH-m) (*n* = 23),Group H. Orthodontic self-drilling mini-implants placement in the molar sector by an augmented reality device (Hololens2, Redmond, WA, USA)based on navigation guidance (AR SCREWS-m) (*n* = 23) and Group I. Orthodontic self-drilling mini-implants placement in the molar sector by conventional freehand technique(FHT-m) (*n* = 23). The teeth assigned to both experimental models presented similar anatomical dimensions evaluated with an electronic caliper and were positioned in the experimental model using a silicone splint to prevent different interradicular spaces between the different teeth of the experimental models.

**Fig. 1:**
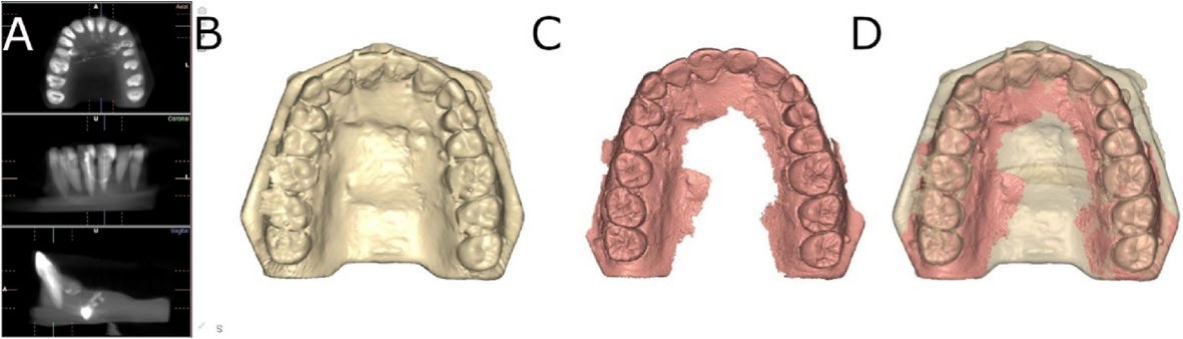
**A** DICOM files from the CBCT scan, **B** render STL digital file from the CBCT scan, **C** STL digital file from the digital impression and **D** alignment procedure between STL and CBCT scan digital files

A preoperative cone-beam computed tomography (CBCT) scan (WhiteFox, Acteón Médico-Dental Ibérica S.A.U.-Satelec, Merignac, France) was taken of the experimental epoxy resin models (Ref. 20–8130-128, EpoxiCure®, Buehler, IL, USA) using the following exposure parameters: 105.0 kV peak, 8.0 milliamperes, 7.20 s, and a field of view of 15 × 13 mm (Fig. 1A, B). A 3D surface scan was subsequently performed via 3D intraoral scan (True Definition, 3 M ESPE ™, Saint Paul, MN, USA) using three-dimensional in-motion video imaging technology (Fig. 1C). The datasets obtained from the digital workflow were added to 3D implant planning software (NemoScan®, Nemotec, Madrid, Spain) to plan the virtual placement of the orthodontic self-drilling mini-implants (Ref. 16-G2-008, Dual Top® Anchor System, JEIL Medical Corporation, Guro-gu, Seoul, Republic of Korea). The orthodontic self-drilling mini-implants were 1.3 mm in diameter, 8.0 mm in length in the active part and 2.0 mm in the inactive part. Virtual placement was planned by matching the three-dimensional surface scan with data from the CBCT, with the key points being overlaid on the crown of the teeth (Fig. 1D). Virtual orthodontic self-drilling mini-implants were placed to a depth of 6 mm, an insertion angle of 90° to the longitudinal axis of the teeth, and a depth of 6.0 mm with respect to the cortical plate.

The interradicular spaces where the orthodontic self-drilling mini-implants were placed were also randomly selected (Epidat 4.1, Galicia, Spain). The experimental procedure was performed according to the methods conducted in a previous study [22].

### Augmented reality technique based on tooth visualization

**Fig. 2:**
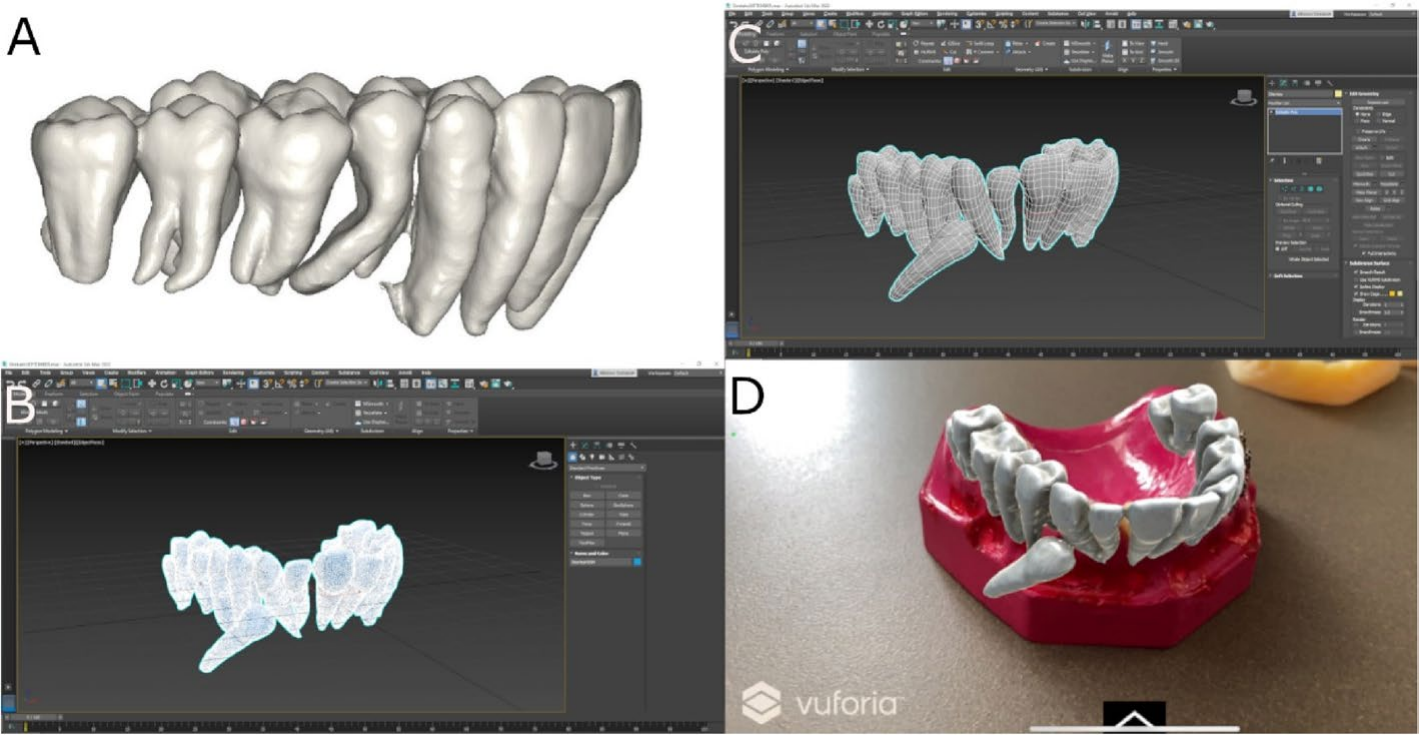
**A** Unified STL digital file after teeth segmentation (**B**, **C**)planning process in augmented reality device software and (**D**) illustration of the STL digital file of the segmented teeth virtually aligned on the experimental model

Furthermore, the DICOM files from the pre-operative CBCT scan (WhiteFox, Acteón Médico-Dental Ibérica S.A.U.-Satelec, Merignac, France) and the STL digital file from the digital impression (True Definition, 3 M ESPE ™, Saint Paul, MN, USA) were uploaded to the 3D implant planning software (NemoScan®, Nemotec, Madrid, Spain). Then, datasets were aligned, and teeth were individually segmented (Fig. 2A). Finally, the STL digital files of each tooth were unified in a single STL. Afterwards, the STL digital file was exported to a multi-platform augmented reality and mixed reality application development platform (Vuforia, Unity Technologies) to allow the identification, tracking and alignment of the STL digital file of the teeth on the experimental models of epoxy resin, by the recognition of key points (dental cusp) (Fig. 2B, C). Afterwards, the multi-platform augmented reality and mixed reality application development platform (Vuforia, Unity Technologies) was installed in an augmented reality appliance (Hololens2, Redmond, WA, USA): Finally, the STL digital file of the teeth was uploaded in the application to visualize the STL digital file on the orography of the experimental models of epoxy resin (Fig. 2D) (Video illustration: https://www.youtube.com/watch?v=fBxAdiyrYTk).

### Augmented reality technique based on navigation guidance

**Fig. 3:**
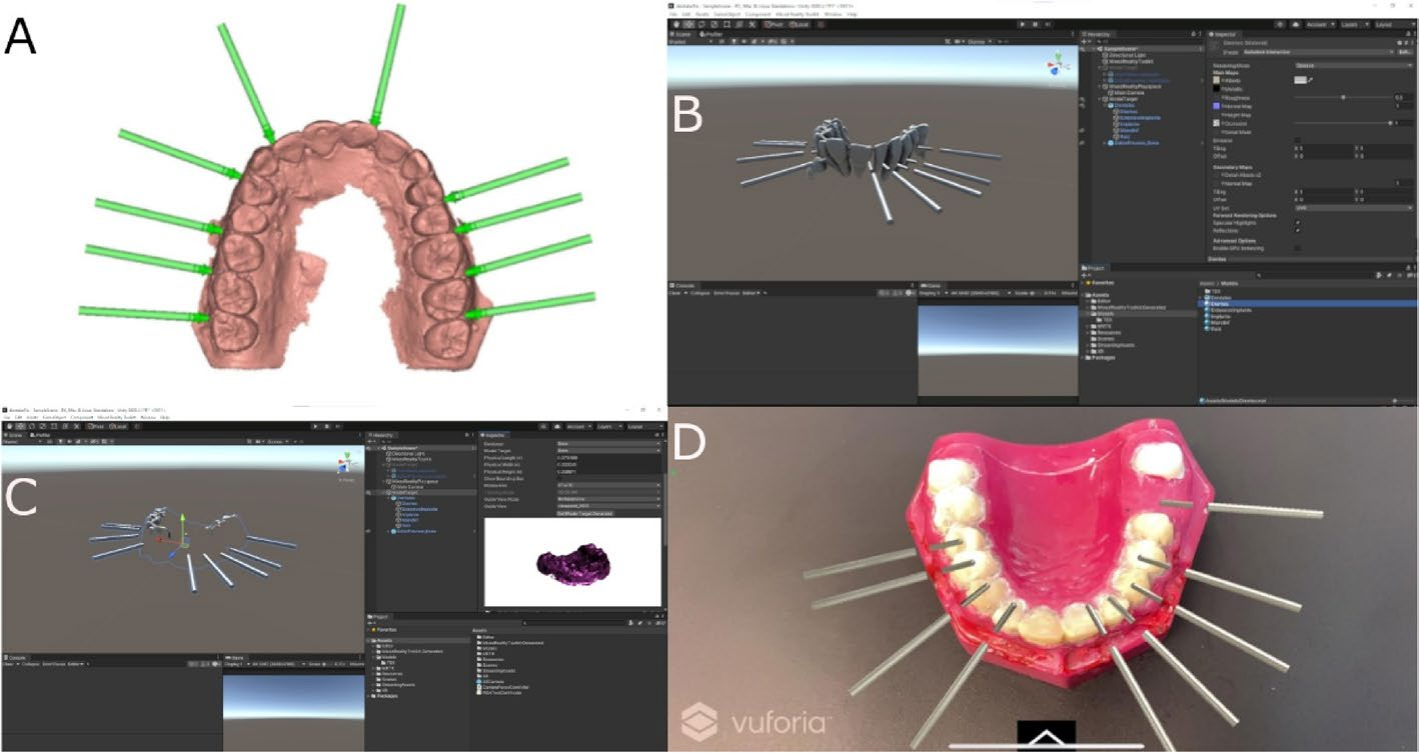
**A** Orthodontic self-drilling mini-implants planned in the 3D implant planning software, **B**,**C** planning process in augmented reality device software and (**D**) illustration of the STL digital file of the orthodontic self-drilling mini-implants navigation guides on the experimental model

Afterwards, the STL digital file of the orthodontic self-drilling mini-implants placement site(Dual Top® Anchor System, JEIL Medical Corporation, Guro-gu, Seoul, Republic of Korea)planned on the 3D implant planning software (NemoScan®, Nemotec, Madrid, Spain) (Fig. 3A) were exported to a multi-platform augmented reality and mixed reality application development platform (Vuforia, Unity Technologies) to allow the identification, tracking and alignment of the STL digital file of the orthodontic self-drilling mini-implants on the experimental models of epoxy resin, by the recognition of key points (dental cusp) (Fig. 3B, C). Afterwards, the multi-platform augmented reality and mixed reality application development platform (Vuforia, Unity Technologies) was installed in an augmented reality appliance (Hololens2, Redmond, WA, USA): Finally, the STL digital file of the orthodontic self-drilling mini-implants was uploaded in the application to visualize the STL digital file on the orography of the experimental models of epoxy resin (Fig. 3D) (Video illustration: https://www.youtube.com/watch?v=fBxAdiyrYTk).

The orthodontic micro-screws (Dual Top® Anchor System, JEIL Medical Corporation, Guro-gu, Seoul, Republic of Korea) randomly assigned to the FHT study group were placed in the experimental models by a unique operator with 10 years’ experience, according to the recommendations performed by Cozzani et al. [22] to place self-tapping orthodontic micro-screws after using a osteotomy pilot drill (Ref.: 112-MC.201, Dual Top® Anchor System, JEIL Medical Corporation, Guro-gu, Seoul, Republic of Korea). All orthodontic micro-screws (Dual Top® Anchor System, JEIL Medical Corporation, Guro-gu, Seoul, Republic of Korea) of AR TOOTH, AR SCREWS and FHT study groups were inserted in the middle of the inter-root space, 2 mm from the alveolar ridge.

### Measurement procedure

**Fig. 4:**
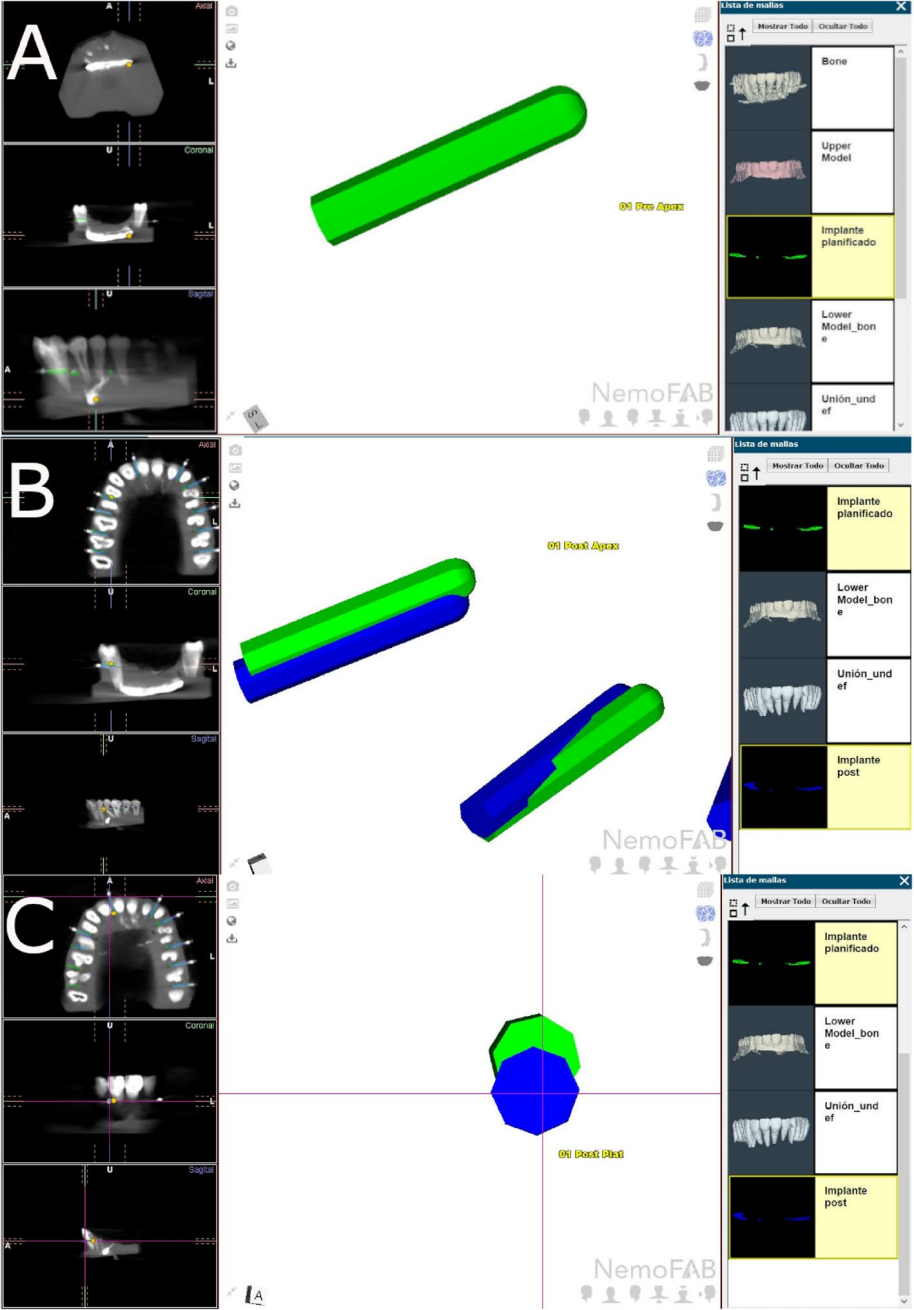
**A** Preoperative position of the orthodontic self-drilling mini-implants, **B** horizontal and **C** angular measurements between preoperative (green cylinder and postoperative (blue cylinder) position of the orthodontic self-drilling mini-implants

After placing the orthodontic self-drilling mini-implants (Dual Top® Anchor System, JEIL Medical Corporation, Guro-gu, Seoul, Republic of Korea), postoperative CBCT scans were taken of the experimental models. Then, then DICOM digital files from the postoperative CBCT scan were uploaded to the 3D implant planning software (NemoScan®, Nemotec, Madrid, Spain). Samely, the STL digital file from the preoperative planning of the orthodontic self-drilling mini-implants (Dual Top® Anchor System, JEIL Medical Corporation, Guro-gu, Seoul, Republic of Korea) location was uploaded to the 3D implant planning software (NemoScan®, Nemotec, Madrid, Spain) (Fig. 4A-C).

**Fig. 5:**
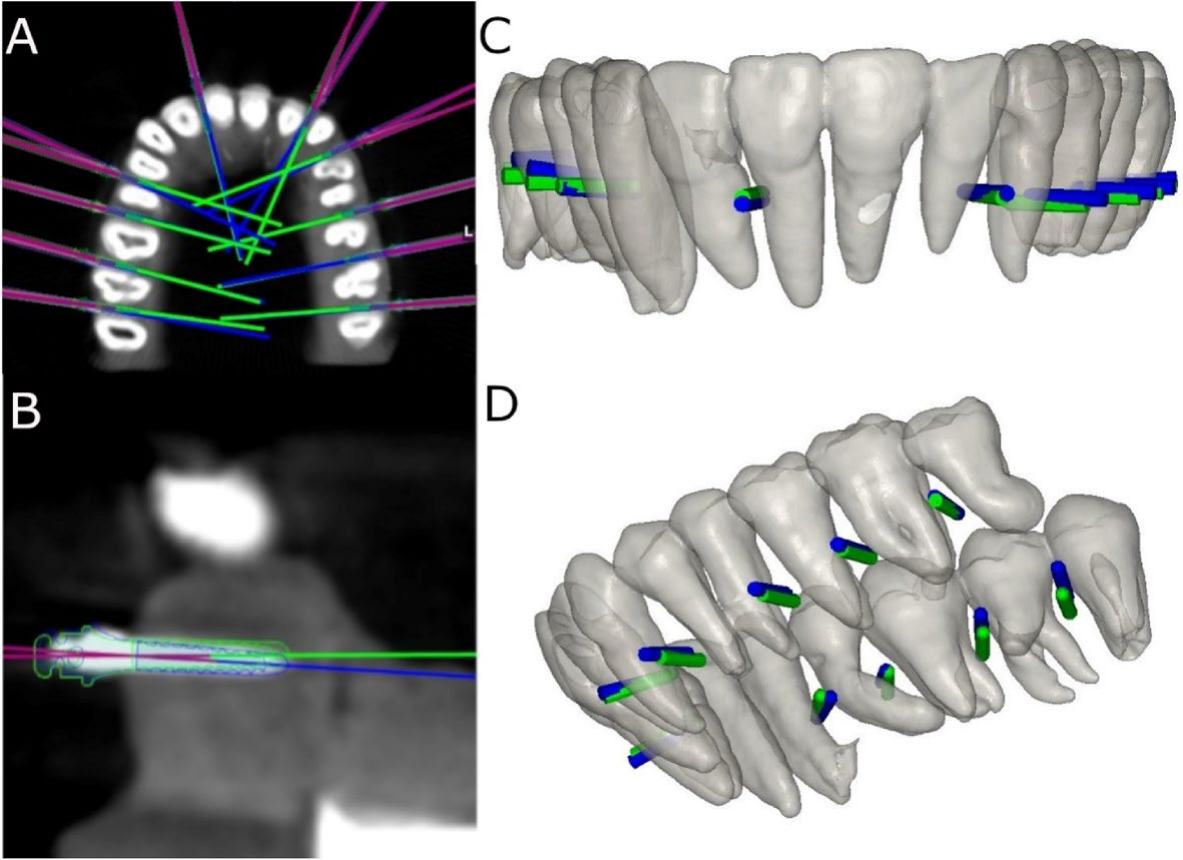
**A**–**D** Deviations measurement procedure between planned (green cylinder) and placed (blue cylinder) orthodontic self-drilling mini-implants in all study groups

Afterwards, the datasets from the postoperative CBCT scan and the STL digital file were then aligned to assess the deviation angle (as measured in the middle of the cylinder) and horizontal deviation (taken at the coronal entry-point and apical end-point of each orthodontic self-drilling mini-implant) between the planning and the postoperative position (Fig. 5A–D) by an independent observer, using a measure tool of the 3D implant planning software (NemoScan®, Nemotec, Madrid, Spain) (Video illustration: https://youtu.be/9ef06jAuDKU). All these experimental procedures were performed according to the methods conducted in a previous study [23].

**Fig. 6:**
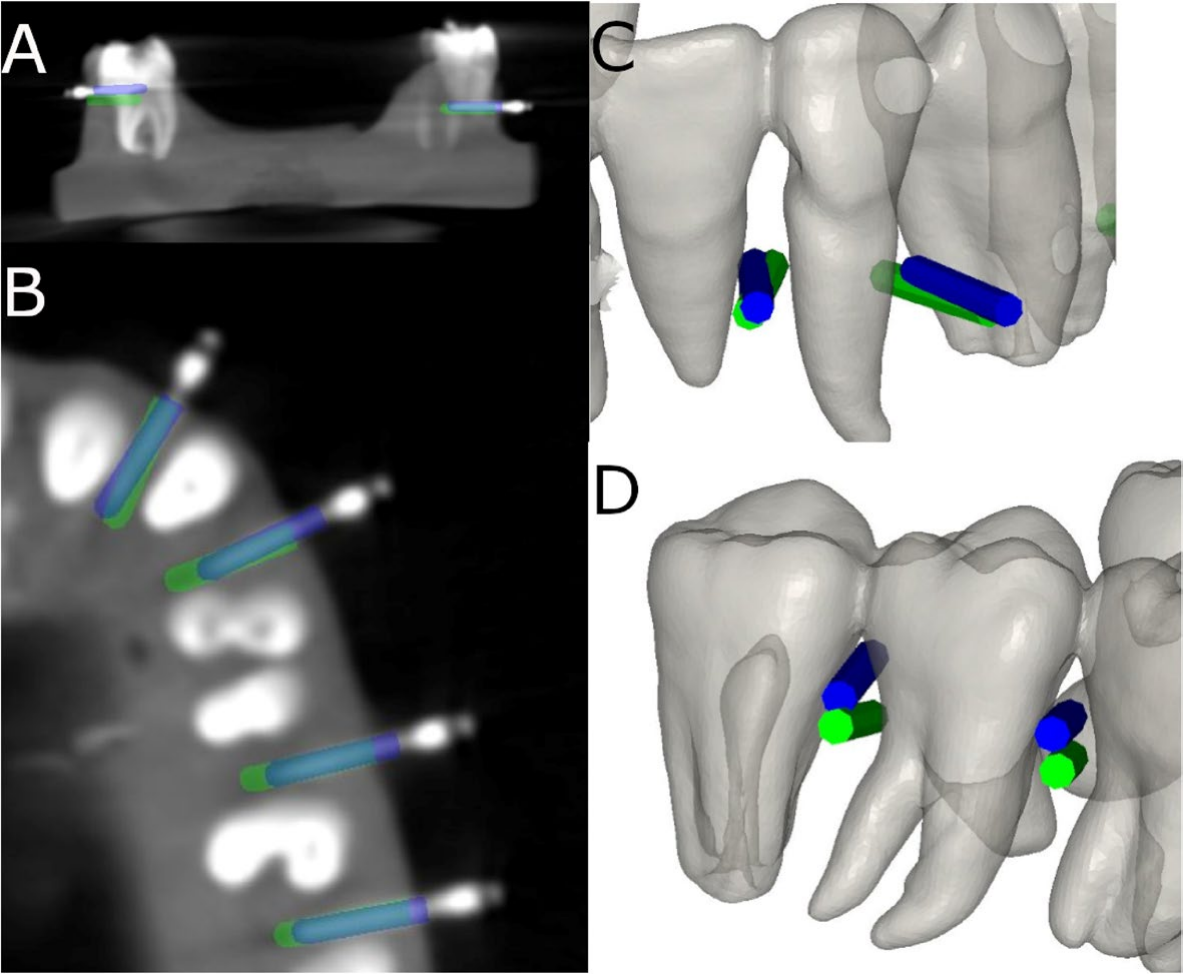
**A** Radiographic analysis of the root perforation in the 3D implant planning software, **B** Relationship between the root processes and the planned (green mini-implants) and performed (blue mini-implants) orthodontic self-drilling mini-implants in all study groups and (**D**) augmented reality technique

Root perforations arising from placement of the orthodontic micro-screws (Dual Top® Anchor System, JEIL Medical Corporation, Guro-gu, Seoul, Republic of Korea) placement were also analyzed and recorded at the 3D implant planning software (NemoScan®, Nemotec, Madrid, Spain) between the conventional freehand technique, mixed reality technique and computer-aided static navigation technique (Fig. 6A–D).

### Reliability test

Reliability between a conventional freehand technique and two navigation techniques based on augmented reality technology for the orthodontic self-drilling mini-implants placement at the coronal entry-point, apical end-point and angular deviation was performed using test–retest statistical analysis. This test consists of administering the same test three times to the same subjects, so that, if it is reliable, the same results will be obtained on both occasions.

Additionally, reliability test was performed Inter-observer reliability was measured using the intraclass correlation coefficient (ICC). This coefficient takes values between 0 and 1, values less than 0.5 indicate low reliability, values between 0.5 and 0.75 moderate reliability, between 0.75 and 0.9 good reliability and reliability from 0.9 excellent [24].

### Statistical tests

All studied variables were recorded using SPSS 22.00 for Windows for statistical analysis. The descriptive statistical analysis used the mean and standard deviation (SD) of quantitative variables. A multivariate (generalized linear model (GLM)) was used for analyzing the effect of the study group, the dental group, and the interaction between the variables in each of the response variables. In case of obtaining a significant result, 2 to 2 tests were carried out a posteriori. To correct the type I error, the *p*-values were corrected using the Tukey correction. As the variables were normally distributed; *p* < 0.05 was determined statistically significant. Reliability was analyzed using the test retest and ICC statistical analysis.

## Results

The means and SD values for coronal entry-point, apical end-point and angular deviation of the conventional freehand technique and two navigation techniques based on augmented reality technology for the orthodontic self-drilling mini-implants placement in all dental sectors are displayed in Table 1.

**Table 1:** Descriptive deviation values at the coronal entry-point (mm), apical end-point (mm), and angular (°) levels of the orthodontic self-drilling mini-implants placed by using conventional freehand technique and two navigation techniques based on augmented reality technology for the orthodontic self-drilling mini-implants placement in all dental sectors

Measure	Study Group	Tooth	N	Mean	SD	Minimum	Maximum
Coronal	AR TOOTH	Incisive-Canine	23	0.59	0.31	0.30	1.30
		Premolar	23	0.61	0.38	0.10	1.40
		Molar	23	1.71	0.30	1.10	2.30
	AR SCREWS	Incisive-Canine	23	1.65	0.57	0.30	2.10
		Premolar	23	1.64	0.65	0.20	2.10
		Molar	23	1.91	0.20	1.30	2.10
	FHT	Incisive-Canine	23	2.28	0.63	1.00	4.00
		Premolar	23	1.70	0.25	1.40	2.10
		Molar	23	2.60	0.65	1.60	3.70
Apical	AR TOOTH	Incisive-Canine	23	0.35	0.30	0.10	0.80
		Premolar	23	0.95	0.17	0.60	1.30
		Molar	23	1.94	0.48	1.30	3.00
	AR SCREWS	Incisive-Canine	23	1.71	0.95	0.00	2.80
		Premolar	23	1.74	0.46	0.80	2.10
		Molar	23	2.12	0.34	1.50	2.80
	FHT	Incisive-Canine	23	0.81	0.34	0.30	1.40
		Premolar	23	1.59	0.35	1.20	2.30
		Molar	23	2.63	0.25	2.20	3.10
Angular	AR TOOTH	Incisive-Canine	23	5.74	4.15	0.40	9.40
		Premolar	23	4.90	3.73	0.00	9.00
		Molar	23	3.90	3.08	1.00	10.00
	AR SCREWS	Incisive-Canine	23	6.08	2.65	2.00	9.80
		Premolar	23	4.89	2.19	0.00	9.00
		Molar	23	5.68	2.47	1.10	9.80
	FHT	Incisive-Canine	23	6.65	1.72	4.10	8.50
		Premolar	23	8.18	4.98	2.30	14.60
		Molar	23	7.91	2.99	4.10	11.50

In addition, comparative analysis according to the orthodontic self-drilling mini-implants placement techniques in all dental sectors are displayed in Table 2.

**Table 2:** Comparative analysis between the orthodontic self-drilling mini-implants placement techniques in all dental sectors

Tooth	Study Group	Study Group	Estimate	SD	*p*-value
Incisive-canine	FHT	MR TOOTH	0.4636	0.1371	0.0025
	FHT	MR SCREWS	-0.9000	0.1371	< .0001
	MR TOOTH	MR SCREWS	-1.3636	0.1371	< .0001
Premolars	FHT	MR TOOTH	0.6455	0.1371	< .0001
	FHT	MR SCREWS	-0.1455	0.1371	0.5395
	MR TOOTH	MR SCREWS	-0.7909	0.1371	< .0001
Molars	FHT	MR TOOTH	0.6913	0.1341	< .0001
	FHT	MR SCREWS	0.5130	0.1341	0.0005
	MR TOOTH	MR SCREWS	-0.1783	0.1341	0.3806

**Fig. 7:**
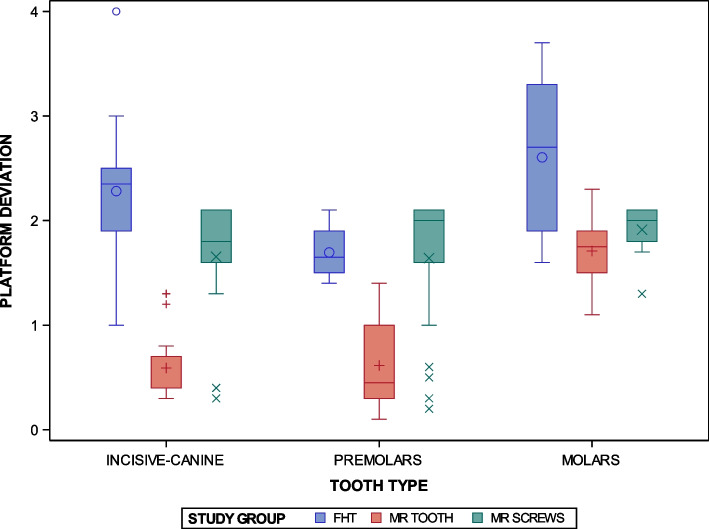
Box plot of the coronal entry-point (mm)deviations in planned and placed orthodontic self-drilling mini-implants placed by using conventional freehand technique and two navigation techniques based on augmented reality technology in all dental sectors. The horizontal line in each box represents the respective median value of the study groups. + ,o, x; Mean value of the box plots

Statistically significant differences were shown between the conventional freehand technique and two navigation techniques based on augmented reality technology for the orthodontic self-drilling mini-implants placement (*p* < 0.0001); specially at the “Incisive-canine” placement site (*p* < 0.0001). Additionally, statistically significant differences were shown between the orthodontic self-drilling mini-implants placement site (*p* < 0.0001); except at the “Premolar” placement site between the AR SCREWS and FHT study groups (*p* = 0.9221) and for the “Molar” placement site between the AR TOOTH and AR SCREWS study groups (*p* = 0.3175). In addition, statistically significant differences were shown between “Premolar” and “Molar” placement sites in the FHT study group (*p* < 0.001), between “Incisive-canine” and “Molar” placement sites in the AR TOOTH study group (*p* < 0.0001), and between “Premolar” and “Molar” placement sites in the AR TOOTH study group (*p* < 0.0001). Moreover, statistically significant differences were also shown at the interaction between the orthodontic self-drilling mini-implants placement techniques and the orthodontic self-drilling mini-implants placement sites were also statistically significant (*p* < 0.0001) (Fig. 7).

**Fig. 8:**
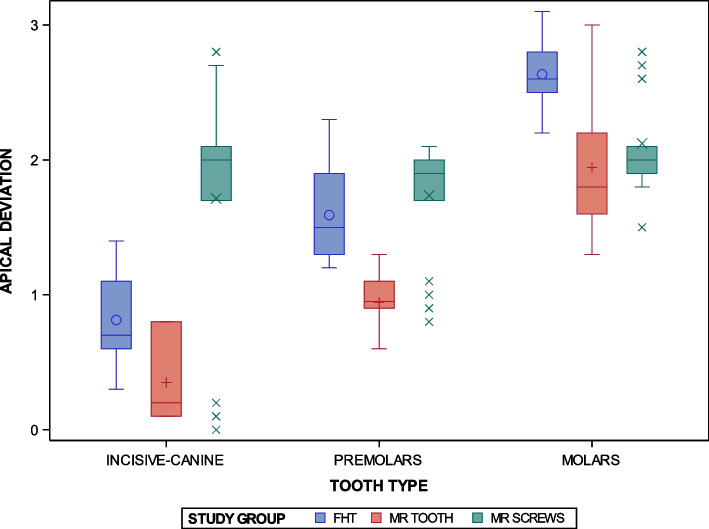
Box plot of the apical end-point (mm) deviations in planned and placed orthodontic self-drilling mini-implants placed by using conventional freehand technique and two navigation techniques based on augmented reality technology in all dental sectors. The horizontal line in each box represents the respective median value of the study groups. + ,o, x; Mean value of the box plots

The paired *t*-test found statistically significant differences between the conventional freehand technique and two navigation techniques based on augmented reality technology for the orthodontic self-drilling mini-implants placement (*p* < 0.0001); specially at the “Incisive-canine” placement site (*p* < 0.0001). Additionally, statistically significant differences were shown between the orthodontic self-drilling mini-implants placement site (*p* < 0.0001); except for the “Incisive-canine” placement site between the FHT and AR TOOTH study groups (*p* = 0.0025), at the “Premolar” placement site between the AR SCREWS and FHT study groups (*p* = 0.5395), and at the “Molar” placement site between the AR TOOTH and AR SCREWS study groups (*p* = 0.3806). In addition, statistically significant differences were shown between all orthodontic self-drilling mini-implants placement sites in the FHT study group (*p* < 0.0001), AR TOOTH study group (*p* < 0.0001), and between the “Incisive-canine” and “Molar” placement site in the AR SCREWS study group; however, no statistically significant differences were shown between the “Incisive-canine” and “Premolar” placement site in the AR SCREWS (*p* = 0.9850), and between the “Premolar” and “Molar” placement site in the AR SCREWS (*p* = 0.0137). Moreover, statistically significant differences were also shown at the interaction between the orthodontic self-drilling mini-implants placement techniques and the orthodontic self-drilling mini-implants placement sites were also statistically significant (*p* < 0.0001) (Fig. 8).

**Fig. 9:**
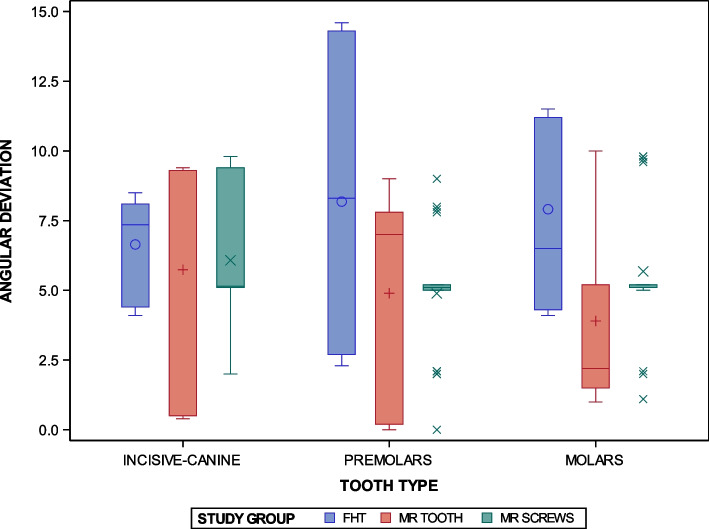
Box plot of the coronal entry-point (mm) deviations in planned and placed orthodontic self-drilling mini-implants placed by using conventional freehand technique and two navigation techniques based on augmented reality technology in all dental sectors. The horizontal line in each box represents the respective median value of the study groups. + ,o, x; Mean value of the box plots

The paired *t*-test found statistically significant differences between the conventional freehand technique and two navigation techniques based on augmented reality technology for the orthodontic self-drilling mini-implants placement (*p* < 0.0001); specifically in the “Premolar” placement site between the FHT and AR TOOTH study groups (*p* = 0.0027), and the FHT and AR SCREWS study groups (*p* = 0.0027), and in the “Molar” placement site between the FHT and AR TOOTH study groups (*p* < 0.0001). However, no statistically significant differences were shown between the orthodontic self-drilling mini-implants placement site (*p* < 0.8469) in the FHT study group (*p* = 0.2477), AR TOOTH study group (*p* = 0.1690) and the AR SCREWS study group (*p* = 0.4688). Finally, statistically significant differences were not shown at the interaction between the orthodontic self-drilling mini-implants placement techniques and the orthodontic self-drilling mini-implants placement sites were also statistically significant (*p* = 0.1126) (Fig. 9).

Eight root perforations were observed in the conventional freehand technique study group after the orthodontic self-drilling mini-implants placement at teeth 1.4, 1.5, 1.6, 1.8, 2.5, 2.6, 2.7 and 2.8, which match with the highest coronal entry-point and apical end-point deviation values. No root perforations were identified in the two navigation techniques based on augmented reality technology.

Correlations between the three measurements performed by both operators showed a *p* = 1; therefore, it can be concluded that the measurements are reliable (Table 3).

**Table 3:** Correlations between the three measurements performed by both operators

Pearson Correlation Coefficients (*p*-value)			
	Result 1	Result 2	Result 3
Result 1	1.00000	1.00000 (< .0001)	1.00000 (< .0001)
Result 2		1.00000	1.00000 (< .0001)
Result 3			1.00000

Additionally, an ICC of 1 was obtained, so the reliability between operators is perfect

## Discussion

The results of the present study reject the null hypothesis (H_0_) that posits that there is no difference in the accuracy and root contact prevalence between a conventional freehand technique and two navigation techniques based on augmented reality technology for the orthodontic self-drilling mini-implants placement at the coronal entry-point, apical end-point and angular deviation in all dental sectors.

Taking into consideration the fact that augmented reality is novel navigation method, it is of great importance to compare it to the conventional methods used for implant positioning in the past, prior to apply it in the clinical practice. The present study showed higher deviations for the conventional freehand technique than both AR navigation techniques at the coronal entry point, apical end-point and angular deviations. These results agree with the ones of Kivovics et al. [25] where the authors compared AR technique with freehanded technique. They found that the coronal and apical deviations in the AR group were significantly lower than those in the free-hand group (coronal deviation of 1.27 ± 0.40 mm, apical deviation of 1.34 ± 0.41 mm and coronal deviation, 1.93 ± 0.79, apical deviation, 2.28 ± 0.74 respectively) but, regarding angular deviation, no significant differences were found between the AR and free-hand groups(angular deviation of 4.09 ± 2.79 and 5.85 ± 2.60° respectively).

In addition, in their in vitro study, Jiang et al. observed that there was an angular deviation of 5.04 ± 2.83° during implant placement using AR navigation [26] and found that AR showed smaller horizontal, vertical, and angular errors in the apical areas of the central incisor and the canine region and provided significantly shorter surgical time than the two-dimensional (2D) image-guided navigation method (*p* < 0.05). Pellegrino et al. [27] in their pilot study of two cases used AR technology in order to place implants in the premolar area. Their results stated 0.53 mm deviation at the entry point and 0.50 mm at the apical point for the first implant and 0.46 mm at the entry point and 0.48 mm at the apical point for the second implant. The angular deviations were respectively 3.05° and 2.19°.

Furthermore Lin et al. [28] in their in vitro study found that deviation of implant placement from planned position was significantly reduced when surgical template and augmented reality technology were used with mean deviations in entry point, apex and angle to be 0.50 ± 0.33 mm, 0.96 ± 0.36 mm, 2.70 ± 1.55° respectively. Similarly, Ma et al. [29] compared in vitro AR-guided dental implant placement and freehand technique and the results agree with the above. The AR technique was more accurate than dentist’s experience (mean target error = 1.25 mm vs. 1.63 mm; mean angle error = 4.03° vs. 6.10°). Finally, in their pig cadaver study, Katić et al. [30] achieved less than 2.5 mm deviation while placing implants using AR-based navigation, and on the same time AR made the surgery easier and showing ergonomic benefits.

Orthodontic micro-screws placement between contiguous roots necessitates proper planning, to determinate the safest placement site and avoid causing damage to the roots or the adjacent structures [16].

Motoyosi et al., categorizes the root proximity of orthodontic micro-screws into three groups, A. no contact between root and orthodontic micro-screw, B. one point of contact between root and the orthodontic micro-screws and C. two or more points of contact [31]. In severe cases of root perforation loss of pulp vitality, ankylosis or root resorption can be caused, although those are rare complications. To assume, the risk of root pathology increases rapidly when orthodontic micro-screws are placed closer to the dental root surface, while the critical proximity found to be 1 mm. Those reasons make accurate position of the orthodontic micro-screws an important parameter of the treatment, as except from tissue damage, deviation of mini screw position can cause loss of its stability [32]. The placement angle although it’s widely accepted that influences a lot the stability; it remains a controversial topic. A finite element analysis of miniscrew angle placement has proved that 90 degrees insertion into 1 mm of cortical bone and 10 mm of trabecular bone provides better anchorage at any direction of force and as a result might increase the stability of the miniscrews [33].

The present study consist of an in vitro study and this has some limitations, because in vitro the insertion of miniscrew is much easier than in a real patient. Also, the number of miniimplants is relatively small especially when it is subdivided into categories according to placement position in each technique. Despite those limitations, the study achieves to concentrate enough points, comparing three different techniques and different miniscrew position sites, which is missing from the literature especially considering the rise of new technologies in dentistry over the past few years.

Finally augmented reality technology is an innovative approach in dental surgery especially in the field of dental implants, but more research should be conducted in order to establish its use in clinical practice.

## Conclusions

- The navigation techniques based on augmented reality technology has an effect on the accuracy of orthodontic self-drilling mini-implants placement and results in fewer intraoperative complications, comparing to the conventional free-hand technique.
- The AR TOOTH augmented reality technique showed more accurate results between planned and placed orthodontic self-drilling mini-implants, comparing to the AR SCREWS and conventional free-hand techniques.
- The navigation techniques based on augmented reality technology showed fewer intraoperative complications, comparing to the conventional free-hand technique.

## Data Availability

The datasets used and/or analyzed during the current study are available from the corresponding author on reasonable request.
